# The effect of Activin‐A on periodontal ligament fibroblasts‐mediated osteoclast formation in healthy donors and in patients with fibrodysplasia ossificans progressiva

**DOI:** 10.1002/jcp.27693

**Published:** 2018-11-11

**Authors:** Ton Schoenmaker, Fenne Wouters, Dimitra Micha, Tim Forouzanfar, Coen Netelenbos, E. Marelise W. Eekhoff, Nathalie Bravenboer, Teun J. de Vries

**Affiliations:** ^1^ Department of Periodontology Academic Centre for Dentistry Amsterdam (ACTA), University of Amsterdam and Vrije Universiteit Amsterdam The Netherlands; ^2^ Department of Clinical Genetics VU University Medical Center, Amsterdam Movement Sciences Amsterdam The Netherlands; ^3^ Department of Oral and Maxillofacial Surgery and Oral Pathology VU University Medical Center, Academic Centre for Dentistry Amsterdam (ACTA), University of Amsterdam and Vrije Universiteit Amsterdam The Netherlands; ^4^ Internal Medicine, Endocrinology Section, VU University Medical Center Amsterdam The Netherlands; ^5^ Department of Clinical Chemistry VU University Medical Center Amsterdam The Netherlands

**Keywords:** Activin‐A, fibrodysplasia ossificans progressiva (FOP), osteoclastogenesis, osteogenesis, periodontal ligament fibroblasts (PLF)

## Abstract

Fibrodysplasia ossificans progressiva (FOP) is a genetic disease characterized by heterotopic ossification (HO). The disease is caused by a mutation in the activin receptor type 1 (*ACVR1*) gene that enhances this receptor's responsiveness to Activin‐A. Binding of Activin‐A to the mutated ACVR1 receptor induces osteogenic differentiation. Whether Activin‐A also affects osteoclast formation in FOP is not known. Therefore we investigated its effect on the osteoclastogenesis‐inducing potential of periodontal ligament fibroblasts (PLF) from teeth of healthy controls and patients with FOP. We used western blot analysis of phosphorylated SMAD3 (pSMAD3) and quantitative polymerase chain reaction to assess the effect of Activin‐A on the PLF. PLF‐induced osteoclast formation and gene expression were studied by coculturing control and FOP PLF with CD14‐positive osteoclast precursor cells from healthy donors. Osteoclast formation was also assessed in control CD14 cultures stimulated by macrophage colony‐stimulating factor (M‐CSF) and receptor activator of nuclear factor kappa‐B ligand (RANK‐L). Although Activin‐A increased activation of the pSMAD3 pathway in both control and FOP PLF, it increased ACVR1, FK binding protein 12 (FKBP12), an inhibitor of DNA binding 1 protein (ID‐1) expression only in FOP PLF. Activin‐A inhibited PLF mediated osteoclast formation albeit only significantly when induced by FOP PLF. In these cocultures, it reduced M‐CSF and dendritic cell‐specific transmembrane protein (DC‐STAMP) expression. Activin‐A also inhibited osteoclast formation in M‐CSF and RANK‐L mediated monocultures of CD14+ cells by inhibiting their proliferation.

This study brings new insight on the role of Activin A in osteoclast formation, which may further add to understanding FOP pathophysiology; in addition to the known Activin‐A‐mediated HO, this study shows that Activin‐A may also inhibit osteoclast formation, thereby further promoting HO formation.

## 1 INTRODUCTION

Fibrodysplasia ossificans progressiva (FOP) is a severe genetic disease, characterized by progressive heterotopic bone formation by which muscles, tendons, and ligaments are converted into bone (Bravenboer et al., 2015; Kaplan et al., 2008; Pignolo, Shore, & Kaplan, 2011). Heterotopic ossification (HO) in FOP appears in flare‐ups during inflammation, following injury or spontaneously. The newly formed extra‐skeletal bone ultimately connects to the existing skeleton, hereby gradually causing irreversible movement impairments throughout the body. In addition, mobility limitations of the thorax often result in a shortened lifespan due to respiratory problems (Kaplan et al., 2010). The worldwide prevalence of FOP is approximately 1 in 2 million people. There is no cure for FOP, current disease management and treatment predominantly consists of prevention and corticosteroids to counteract flare‐ups (Di Rocco et al., 2017; Pignolo, Shore, & Kaplan, 2013).

FOP is caused by mutations in the gene encoding the Activin receptor type 1 (ACVR1)/activin kinase 2 bone morphogenetic protein (BMP) type 1 receptor, most frequently by the single nucleotide 617G > A mutation. This results in the replacement of the amino acid arginine by histidine (R206H; Pignolo et al., 2011; Shore et al., 2006). In healthy subjects, ACVR1 receptor dimers stimulate the SMAD1/5/8 pathway and subsequent osteogenesis by forming a complex with a BMP type 2‐receptor dimer upon binding of different ligands of the tranforming growth factor beta (TGF‐β) superfamily. The ACVR1‐R206H mutation causes decreased binding of the ACVR1‐inhibitor FK binding protein 12 (FKBP12), resulting in leaky signaling of the receptor in the absence of ligand (Billings et al., 2008; Song et al., 2010). Also, various reports have shown altered BMP signaling and increased responsiveness to BMP4 (de la Pena et al., 2005; Fiori, Billings, de la Pena, Kaplan, & Shore, 2006; Kaplan et al., 2006). Recently it has been shown that the mutation also results in an increased responsiveness of the receptor to Activin‐A (Hatsell et al., 2015; Hino et al., 2015). This TGF‐β superfamily ligand is known to induce SMAD2/3 signaling (Pearsall et al., 2008), and normally inhibits BMP signaling through ACVR1 (Olsen et al., 2015). However, in FOP patient‐derived induced pluripotent stem cells (Hino et al., 2015) and in a mouse model of FOP (Hatsell et al., 2015), Activin‐A was specifically shown to signal through the canonical BMP‐pSMAD1/5/8 pathway, thus stimulating osteogenesis. Activin‐A is upregulated during inflammation (Protic et al., 2017), a state that is able to induce flare‐ups and HO in patients with FOP. In a mouse model for FOP, antibodies against Activin‐A resulted in complete inhibition of HO (Hatsell et al., 2015).

Bone is a highly dynamic tissue which is constantly remodeled. Bone remodeling is the continuous process of bone formation (osteogenesis) by osteoblasts, and bone resorption by multinucleated osteoclasts, that form through the fusion of CD14+ monocytes from blood (Sorensen et al., 2007). In healthy bone tissue, there is a balance between bone formation and bone resorption (Florencio‐Silva, Sasso, Sasso‐Cerri, Simoes, & Cerri, 2015). The metabolism, structure, and composition of heterotopic bone formed in patients with FOP are assumed to be comparable with healthy bone (Kaplan et al., 2008). During the active phase of HO formation under influence of Activin‐A however, bone resorption activity is likely reduced, ultimately resulting in the formation of extra, heterotopic bone. Therefore, in this study, we aimed to investigate the role of Activin‐A in osteoclast formation in FOP.

Little is known about the effect of Activin‐A on osteoclast formation from human CD14+ osteoclast precursor cells. In vivo, the necessary signals for osteoclast formation are derived from osteoblast‐like stromal cells (Florencio‐Silva et al., 2015). In the current study, periodontal ligament fibroblasts (PLF) are used as osteoclast formation‐stimulating cells. These cells reside in the periodontal ligament between the root surface of the tooth and the alveolar bone and exhibit both bone forming osteogenic‐ (Arceo, Sauk, Moehring, Foster, & Somerman, 1991; Choi, Noh, Park, Lee, & Suh, 2011; Ruppeka‐Rupeika et al., 2018) and osteoclastogenesis‐inducing (de Vries et al., 2006; Sokos, Everts, & de Vries, 2015) capacities, the latter in combination with osteoclast precursor cells.

The use of tooth‐related cells as a cell model is especially relevant in FOP studies. Bone biopsies, as a source for osteoblastic cells, cannot be taken in patients with FOP because of the risk of heterotopic bone formation as a result of the surgical trauma. After tooth extraction, however, no HO occurs at sites of extraction. Billings et al. (2008) and Wang, Shore, Pignolo, and Kaplan (2017) used cells from discarded primary teeth (stem cells from human exfoliated decidious teeth cells) from patients with FOP showing higher osteogenic differentiation which was shown to be driven by the FOP mutation restricted induction by Activin‐A. We have recently reported the use of PLF as a tool to study osteoclastogenesis in FOP (de Vries et al., 2018), where a stronger inhibitory effect of TGF‐β inhibitor on osteoclast formation was observed in FOP fibroblasts‐mediated osteoclast formation. Here we use this model to study the effect of Activin‐A on osteoclastogenesis driven by FOP PLF. We hypothesize that Activin‐A reduces the osteoclastogenesis‐inducing capacity of the PLF that express the mutant R206H ACVR1 resulting in less bone resorption.

## 2 MATERIALS AND METHODS

### 2.1 Cells

#### 2.1.1 Periodontal ligament fibroblasts

Periodontal ligament cells were retrieved from four extracted third molars from a female patient with FOP aged early 20s, three molars from a female patient with FOP aged early 40s and six third molars from five controls (two males, three females, age range: 15–24). Both patients with FOP carried the classical R206H mutation. Written informed consent was obtained from each participant. Researchers were not able to trace the origin of the material to the person, as required by Dutch law. There were no differences in bone healing between the patients with FOP and the control group after tooth removal. Periodontal ligament was scraped off the middle one third of the root and cells were propagated in culture medium, consisting of Dulbecco's minimal essential medium (Gibco BRL, Paisley, Scotland) supplemented with 10% fetal calf serum (FCS) (HyClone, Logan, UT), and 1% antibiotics: 100 U/ml penicillin, 100 µg/ml streptomycin, and 250 ng/ml amphotericin B (Sigma, St. Louis, MO). Cells were propagated and third passage cells were frozen and stored in liquid nitrogen. All experiments were performed with fourth passage cells.

#### 2.1.2 CD14+ cells

CD14^+^ monocytes were isolated as described before (ten Harkel et al., 2015). Briefly, peripheral blood mononuclear cells were isolated from human buffy coats from healthy donors (Sanquin, The Netherlands) using Ficoll‐Paque density gradient centrifugation. Subsequently, cells were incubated with CD14‐antibody tagged microbeads (Miltenyi Biotec, Bergisch Gladbach, Germany) and sorted using a manual MACS magnetic cell sorter (Miltenyi Biotec) according to the manufacturer's instructions (Davison et al., 2014). The purity of the cells was determined with flow cytometry (FACSverse^™^; BD Biosciences, Piscataway). For analysis, cells were incubated with fluoresceine isothiocyanate (FITC) labeled anti‐human CD14 (Miltenyi Biotec) or its equivalent isotype control IgG2a (Miltenyi Biotec), and incubated for 30 min in the dark on ice. After incubation, cells were washed to remove unbound antibodies, recovered in FACS buffer and analyzed (30 s or 100,000 viable events) on a BD Bioscience FACSverse flow cytometer. Purity was confirmed to be at least 80%.

### 2.2 Osteoclastogenesis

#### 2.2.1 In PLF and CD14+ cells coculture

For the coculture experiments, PLF were cultured overnight in a 48 wells plate at a density of 1.5 × 10^4^ cells/well. Cells were seeded both on plastic and on bovine cortical bone slices of 650 μm thickness which were soaked in a 48 well plate with 400 μl culture medium for 2 hr before seeding. The next day purified CD14+ cells were seeded on top of the PLF at a density of 1 × 10^5^ cells/well and the cells were cultured without or with 50 ng/ml Activin‐A (Sigma).

The cocultures were performed in minimal essential medium alpha modification (αMEM) supplemented with 10% FCS and 1% antibiotics at 37°C, in a humidified atmosphere under 5% CO_2._ Culture media were replaced every 3–4 days.

Six different PLF cell isolates from both control and FOP donors were cocultured with CD14+ cells isolated from one buffy coat. The different experimental conditions were cultured in duplicate. Cells were cultured for 7 days (for RNA isolation) and 21 days (for RNA isolation and tartrate‐resistant acid phosphatase [TRAcP] staining).

#### 2.2.2 In CD14+ cells monoculture

Purified CD14+ cells were suspended in culture medium consisting of αMEM supplemented with 10% FCS, and 1% antibiotics: 100 U/ml penicillin, 100 µg/ml streptomycin, and 250 ng/ml amphotericin B. Cells were cultured in a 96 well plate at a density of 1 × 10^5^ cells/well, for the first three days with macrophage colony‐stimulating factor (M‐CSF; R&D systems, Oxon, UK) 25 ng/ml, without or with 50 ng/ml Activin‐A. After three days the medium composition was changed to M‐CSF 10 ng/ml and receptor activator of nuclear factor kappa‐B ligand (RANKL; R&D Systems) 2 ng/ml, without or with 50 ng/ml Activin‐A. All cultures were maintained at 37°C, in a humidified atmosphere under 5% CO_2._ Culture media were replaced every 3–4 days.

The different experimental conditions were cultured in triplicate. The effect of Activin A on CD14+ cell and cytokines M‐CSF and RANKL‐driven osteoclast formation was re‐established in three different experiments using control CD14+ cells from four healthy volunteers.

Cells were cultured for 7 days (DNA measurement), 14 days (TRAcP staining, DNA measurement) and 21 days (TRAcP staining).

Activin‐A was measured with the Human Activin‐A enzyme‐linked immunosorbent assay kit (Abcam, Cambridge, UK). No detectable Activin‐A (sensitivity < 12 pg/ml) was found in the culture media, nor in the supernatants after 3 days of culturing (data not shown).

### 2.3 Western blot analysis

For the western blot analysis, PLF was cultured in 6 well plates, 3 × 10^5^ cells/well. After overnight culture under serum‐free conditions, cells were incubated without or with 50 ng/ml Activin‐A for 1 hr. Whole cell lysate preparation and western blot analysis was performed as described before (Micha et al., 2016). Shortly, cells were lysed using the NuPAGE LDS Sample Buffer with the NuPAGE reducing agent (Thermo Fisher Scientific, Waltham, MA). Proteins were separated on NuPAGE 4–12% BT gels and were subsequently transferred to nitrocellulose membranes using the iBlot Dry Blotting system (Thermo Fisher Scientific). Immunoblotting was performed overnight with primary antibodies against phosphoSmad3 (Abcam, Cat#ab52903) and actin (Abcam, Cat#ab14128). Secondary antibody incubation was carried out for 1 hr with the IRDye 800CW goat anti‐rabbit IgG and the IRDye 680CW goat anti‐mouse IgG antibodies (LI‐COR Biosciences, Lincoln, NE). Fluorescence was visualized and quantified by the Odyssey infrared imaging system equipped with the Odyssey version 4 software (LI‐COR Biosciences).

### 2.4 TRACP staining and osteoclast quantification

TRACP staining was performed with the Leukocyte Acid Phosphatase Staining Kit (Sigma) as previously described (de Vries et al., 2006). Nuclei were stained with diamidino‐2 phenylindole dihydrochloride (DAPI). Micrographs of cultures on plastic were taken from five fixed positions per well, with a digital camera (Leica, Wetzlar, Germany) and analyzed for the number of TRACP+multinucleated cells (MNCs) containing three or more nuclei. For the cultures on the bone, the total number of TRACP+MNCs on the complete bone slice was counted under the microscope and corrected for the area of the bone slice. Each experimental condition was assessed in duplicate for the cocultures and in triplicate for the CD14+ monocultures.

### 2.5 RNA isolation and real‐time quantitative polymerase chain reaction

For the RNA isolation cells were cultured under different conditions; PLF were cultured in a 48 wells plate, 1.5 × 10^4^ cells/well, samples were taken after 24 hr incubation without or with 50 ng/ml Activin‐A (four wells were pooled per condition). For the coculture 1.5 × 10^4^ PLF were cultured together with 1 × 10^5^ CD14+ cells in a 48 wells plate without or with 50 ng/ml Activin‐A, samples were taken after 7 and 21 days of culture (two wells were pooled per condition).

RNA was isolated using the RNeasy mini kit (Qiagen, Hilden, Germany) following the manufacturer's instructions. The reverse transcriptase reaction was performed with the first strand complementary DNA (cDNA) synthesis kit (Thermo Fisher Scientific) according to the manufacturer's protocol, using both the Oligo(dT)18 and the D(N)6 primers.

Quantitative polymerase chain reaction (Q‐PCR) primers were designed using Primer Express software, version 2.0 (Applied Biosystems, Foster City, CA; Table 1). To avoid amplification of genomic DNA, each amplicon spanned at least one exon‐exon junction. Q‐PCR was performed with the LC480 light cycler (Roche, Basel, Switzerland). Three nanogram cDNA was used in a total volume of 20 µl containing Light Cycler SybrGreen1 Master mix (Roche) and 1 µM of each primer. Tm for all primers is listed in Table 1. In the PLF monocultures hypoxanthine phosphoribosyltransferase 1 (*HPRT1*) was used as housekeeping gene. In the cocultures, porphobilinogen deaminase (*PBGD*) was used. Expression of housekeeping genes was not affected by the different experimental conditions. Samples were normalized based on the expression of the housekeeping gene by calculating the Δ*Ct* (*Ct*_,gene of interest_ − *Ct*_,housekeeping gene_) and expression of the different genes was expressed as 2^−(Δ*Ct*)^. All qPCRs had equal efficiencies

**Table 1:** Primers used for quantitative PCR

Gene	Sequence 5′‐3′	Amplicon length (bp)	Ensemble Gene ID
*HPRT1*	TgACCTTgATTTATTTTgCATACC	101	ENSG00000165704
	CgAgCAAgACgTTCAgTCCT		
*PBGD*	TgCAgTTTgAAATCATTgCTATgTC	84	ENSG00000113721
	AACAggCTTTTCTCTCCAATCTTAgA		
*CSF1*	CCgAggAggTgTCggAgTAC	100	ENSG00000184371
	AATTTggCACgAggTCTCCAT		
	CTCggAgCTCTgATgTgTTgAA		
*DCSTAMP*	ATTTTCTCAgTgAgCAAgCAgTTTC	101	ENSG0000016493
	AgAATCATggATAATATCTTgAgTTCCTT		
*ACVR1*	CAgCTgCCCACTAAAggAAAAT	68	ENSG00000115170
	AATAATgAggCCAACCTCCAAgT		
*FKBP1A/FKBP12*	gATCCgAggCTgggAAgAAg	68	ENSG00000088832
	ggAgATATAgTCAgTTTggCTCTCTgA		
*ID‐1*	ACgTgCTgCTCTACgACATgA	56	ENSG00000125968
	TgggCACCAgCTCCTTgA		

*Note. ACVR1*: Activin A receptor type I; *CFS1*: colony‐stimulating factor1 (coding for macrophage‐colony stimulating factor [M‐CSF]); *DC‐STAMP*: dendritic cell‐specific transmembrane protein; *FKBP1A*: FK506 binding protein 1A (coding for FK binding protein 12 [FKBP12]); *HPRT1*: hypoxanthine phosphoribosyltransferase 1; ID‐1: Inhibitor of DNA binding 1 protein; *PBGD*: porphobilinogen deaminase ; PCR: polymerase chain reaction.

For each gene, the ﬁrst oligonucleotide sequence represents the forward primer, the second sequence represents the reverse primer.

### 2.6 Proliferation assay

Proliferation was assessed by measuring the amount of DNA after 7 and 14 days ofcultureturing using the CyQuant cell proliferation assay kit (Thermo Fisher Scientific) according to the manufacturer's instructions. Briefly, sample lysate was incubated with GR dye reagent (1×) following the manufacturer's instructions. Fluorescence (excitation = 480 nm, emission = 520 nm) was detected using the synergy multi‐mode reader (Biotek, VT).

Each experimental condition was assessed in triplicate.

### 2.7 Statistical analysis

Differences between cultures without and with Activin‐A were tested using the Wilcoxon matched‐pairs signed rank test. For differences between control and FOP cultures the Mann–Whitney test was used. Differences were considered to be significant when the *p*‐value was lower than 0.05.

## 3 RESULTS

### 3.1 Activin‐A induces SMAD 3 phosphorylation in PLF

**Figure 1:**
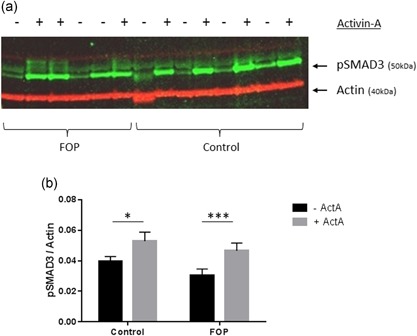
Western blot analysis of SMAD phosphorylation in fibroblasts. Control or FOP PLF were cultured for 1 hr with 50 ng/ml Activin‐A. Whole cell lysates were used for western blot analysis for pSMAD3, actin was used as the loading control (only one of the two gels analyzed is shown) (a). Activin‐A upregulates Smad3 phosphorylation in both control and FOP cells (b). Control *n* = 4, FOP *n* = 6. (Wilcoxon matched‐pairs signed rank test, *p ≤ 0.05, ***p ≤ 0.001). FOP: fibrodysplasia ossificans progressiva; PLF: periodontal ligament fibroblasts; SMAD: mothers against decapentaplegic homolog 3 [Color figure can be viewed at wileyonlinelibrary.com]

To show the induction of the mothers against decapentaplegic homolog 3 (SMAD3) signaling pathway by Activin‐A in PLF, we cultured the cells for 1 hr with Activin‐A. Cell lysates were prepared and western blot analysis was performed with a phospho‐Smad3 antibody. p‐SMAD3 was significantly increased by Activin‐A in both control and FOP cells (Figure 1 a,b).

### 3.2 Activin‐A induces ACVR1, FKBP12, and ID‐1 expression in FOP PLF

**Figure 2:**
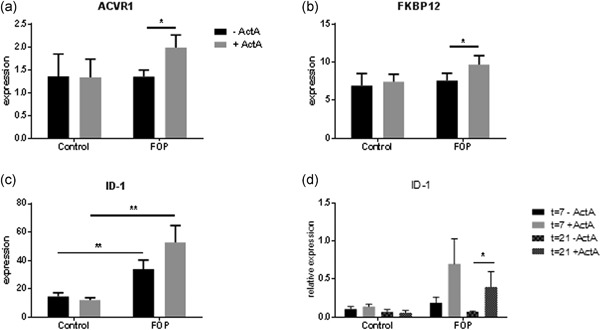
R206H mutation in ACVR1 causes altered responsiveness of the receptor. Control or FOP PLF were cultured for 24 hr without and with Activin‐A 50 ng/ml. Gene expression of ACVR1, FKBP12 and ID‐1 was assessed and normalized for expression of the housekeeping gene HPRT (a, b and c). Both ACVR1 and FKBP12 are significantly upregulated by Activin‐A in FOP PLF (a and b). *n* = 6 for both Control and FOP (Wilcoxon matched‐pairs signed rank test, ^*^ *p* ≤ 0.05). ID‐1 is significantly higher expressed in FOP PLF, both without and with Activin‐A (c). *n* = 6 for both Control and FOP (Mann–Whitney test, ^*^ *p* ≤ 0.05). Control or FOP PLF were cocultured with CD14+ cells from healthy subjects without and with Activin‐A 50 ng/ml. After 7 and 21 days, RNA was isolated and QPCR was performed on ID‐1 and normalized for expression of the housekeeping gene PBGD (d). Activin‐A upregulates ID‐1 expression in cocultures with FOP cells. *n* = 6 for both Control and FOP. (Wilcoxon matched‐pairs signed rank test, ^*^ *p* ≤ 0.05). ACVR1: activin receptor type 1; FKBP12: FK binding protein 12; FOP: fibrodysplasia ossificans progressiva; HPRT: hypoxanthine phosphoribosyltransferase 1; ID‐1: inhibitor of DNA binding 1 protein; PBGD: porphobilinogen deaminase; PLF: periodontal ligament fibroblasts; QPCR: quantitative polymerase chain reaction

To establish whether the R206H mutation indeed results in altered responsiveness of the receptor to Activin‐A, we investigated messenger RNA expression of several genes after a 24 hr incubation of the PLF with Activin‐A. Both the ACVR1 receptor and its inhibitor FKBP12 are significantly upregulated by Activin‐A in the FOP PLF (Figure 2a,b). To test if the SMAD1/5/8 signaling pathway was differentially affected in the FOP PLF we investigated the expression of the inhibitor of DNA binding 1 protein (ID‐1) gene in both the PLF monocultures as well as in the cocultures. ID‐1 is one of the target molecules of SMAD1/5/8 signaling which has been shown to be upregulated by Activin‐A especially in FOP cells (Wang et al., 2017). In the PLF monocultures, ID‐1 was significantly higher expressed in FOP PLF as compared with control PLF both without and with Activin‐A (Figure 2c).

Activin‐A significantly increased ID‐1 expression only in cocultures with the FOP PLF after 21 days. After 7 days of coculture this increase was not significant (*p* = 0.0625; Figure 2d).

### 3.3 Activin‐A inhibits PLF‐induced osteoclast formation

**Figure 3:**
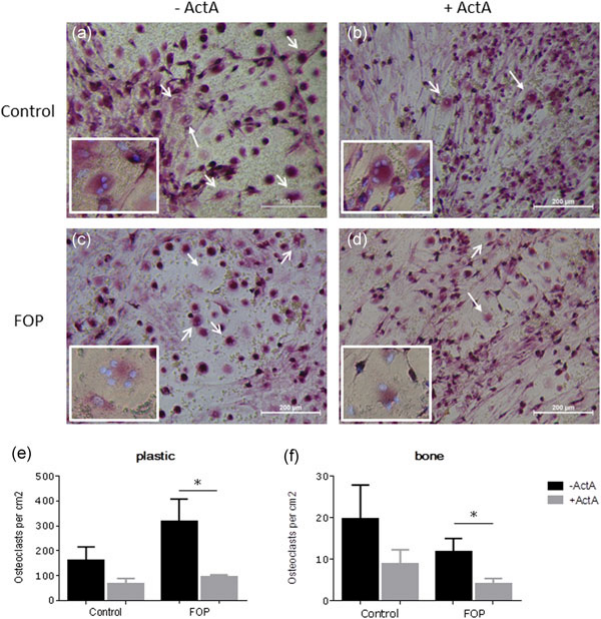
Activin‐A inhibits osteoclast formation in cocultures. Control or FOP PLF were cocultured with CD14+ cells from a healthy subject without and with Activin‐A 50 ng/ml for 21 days on plastic and on bone. Typical micrographs of osteoclast formation on plastic in a coculture of CD14+ cells with (a) control PLF without Activin‐A (b) control PLF with Activin‐A (c) FOP PLF without Activin‐A (d) FOP PLF with Activin‐A are shown, osteoclasts indicated with arrows. Osteoclasts indicated with solid arrowheads are shown at a higher magnification in the left lower corner of the corresponding micrographs. The number of TRAcP positive multinucleated (more than three nuclei) cells were counted on (d) plastic and (e) bone and were conferred to the number of cells/cm^2^. Activin‐A inhibits osteoclast formation in cocultures with FOP PLF. *n* = 6 for both control and FOP (Wilcoxon matched‐pairs signed rank test, ^*^ *p* ≤ 0.05). FOP: fibrodysplasia ossificans progressiva; PLF: periodontal ligament fibroblasts; TRAcP: tartrate resistant acid phosphatase [Color figure can be viewed at wileyonlinelibrary.com]

Having shown the altered responsiveness of the mutated receptor in PLF to Activin‐A, we next assessed whether this alteration also affected their osteoclastogenesis‐inducing capacity. Osteoclasts were differentiated in cocultures of PLF and CD14+ cells without and with Activin‐A both on plastic and on bone. After 21 days the cells were fixed and stained for TRACP and the nuclei were stained with DAPI. As described before, osteoclasts are formed at places were the fibroblasts retract to form cell free areas where the osteoclast precursors can fuse (Bloemen, Schoenmaker, de Vries, & Everts, 2011; Perez‐Amodio, Beertsen, & Everts, 2004). Micrographs show the formation of cell free areas and subsequent formation of osteoclasts in cocultures with control and FOP fibroblasts without and with Activin‐A on plastic (Figure 3a–d). The number of TRACP positive MNCs (with three and more nuclei) was counted (Figure 3e,f). Activin‐A inhibited osteoclast formation in the cocultures of CD14+ cells with both the control as well as the FOP PLF, but only significantly in the cocultures of FOP PLF, both on plastic and on bone.

### 3.4 Activin‐A downregulates genes involved in osteoclast formation

**Figure 4:**
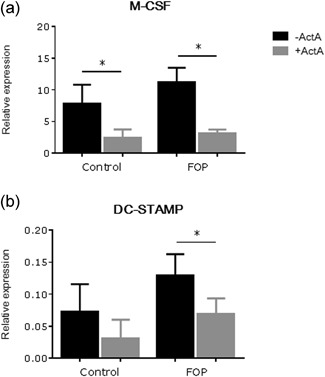
Activin‐A down regulates expression of osteoclast‐ and osteoclastogenesis related genes after 7 days of coculture. Control or FOP PLF were cocultured with CD14+ cells from healthy subjects without and with Activin‐A 50 ng/ml. After 7 days RNA was isolated and QPCR was performed on (a) M‐CSF and (b) DC‐STAMP. Expression was normalized for expression of the housekeeping gene PBGD. *n* = 6 for both control and FOP (Wilcoxon matched‐pairs signed rank test, ^*^ *p* ≤ 0.05). Activin‐A significantly downregulates expression of M‐CSF in the cocultures with the control PLF and of M‐CSF and DC‐STAMP in the cocultures with the FOP PLF. DC‐STAMP: dendritic cell‐specific transmembrane protein; FOP: fibrodysplasia ossificans progressiva; M‐CSF: macrophage colony‐stimulating factor; PBGD: porphobilinogen deaminase; PLF: periodontal ligament fibroblasts; QPCR: quantitative polymerase chain reaction

In an earlier study we already showed by allele‐specific QPCR that the mutated receptor is only expressed by the FOP PLF (de Vries et al., 2018). To find a molecular mechanism underlying the osteoclastogenesis inhibition observed in the cocultures in the presence of Activin‐A, QPCR experiments were performed after 7 days of culturing. In line with the decreased number of osteoclasts that formed, Activin‐A significantly inhibited M‐CSF (Figure 4a) expression in both control and FOP cultures. Furthermore, it lowered the expression of the fusion molecule dendritic cell‐specific transmembrane protein (DC‐STAMP; Figure 4b). This was only significant in the FOP cultures, in line with the significant decrease in osteoclast formation.

### 3.5 Activin‐A inhibits osteoclast formation and activity in CD14+ monocultures

**Figure 5:**
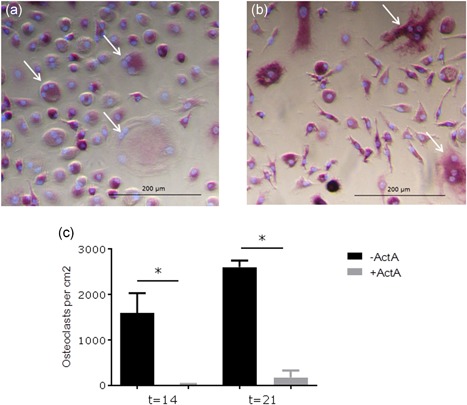
Activin‐A inhibits osteoclastogenesis in CD14+ monocultures. CD14+ cells isolated from a buffy coat from a healthy donor were cultured for 14 and 21 days in the presence of M‐CSF and RANK‐L (a) without, and (b) with 50 ng/ml Activin‐A. The number of TRAcP positive multinucleated cells (more than three nuclei) were counted (c). *n* = 3 (Wilcoxon matched‐pairs signed rank test, ^*^ *p* ≤ 0.05). Activin‐A significantly inhibits osteoclast formation. M‐CSF: macrophage colony‐stimulating factor; RANK‐L: receptor activator of nuclear factor kappa‐B ligand; TRAcP: tartrate resistant acid phosphatase [Color figure can be viewed at wileyonlinelibrary.com]

The above suggests that Activin‐A has an effect on osteoclastogenesis driven by both control as well as FOP patient‐derived PLF, albeit that the effect in the FOP cultures was more pronounced. To investigate whether Activin‐A also directly affected osteoclast precursors, that is in the absence of PLFs, the influence of Activin‐A on osteoclastogenesis in a CD14+ monoculture, derived from healthy subjects, was assessed. Cells were cultured in the presence of M‐CSF and RANK‐L, without and with Activin‐A and osteoclasts were counted after 14 and 21 days. When the CD14+ cells were cultured in the presence of Activin‐A the cells appeared to be more elongated compared to the cultures without Activin‐A (Figure 5a,b). Osteoclasts were formed under both culture conditions (Figure 5a,b, white arrows) and Activin‐A significantly inhibited osteoclastogenesis at both time points (Figure 5c).

### 3.6 Activin‐A decreases proliferation in CD14+ monocultures

**Figure 6:**
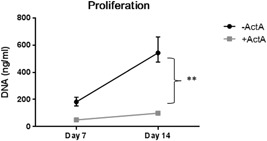
Activin‐A decreases proliferation in CD14+ monocultures. CD14+ cells isolated from a buffy coat from a healthy donor were cultured for 7 and 14 days in the presence of M‐CSF and RANK‐L without and with 50 ng/ml Activin‐A. Proliferation from Day 7 to Day 14 is shown (average increase without Activin‐A 2.98 ± 0.18, with Activin‐A 2.00 ± 0.22, **). *n* = 3 (Wilcoxon matched‐pairs signed rank test, ^**^ *p* ≤ 0.01). Activin‐A significantly inhibits proliferation of CD14+ cells. M‐CSF: macrophage colony‐stimulating factor; RANK‐L: receptor activator of nuclear factor kappa‐B ligand

Because osteoclasts arise through the fusion of precursors, one of the possible reasons for a diminished osteoclast formation could be a decreased proliferation. This was tested by measuring DNA content after 7 and 14 days of culturing in the presence of M‐CSF and RANK‐L, without and with Activin‐A. In both the cultures without and with Activin‐A there was a significant increase in proliferation between Day 7 and 14 (Figure 6). In the cultures with Activin‐A however, this increase was significantly less as compared to the cultures without Activin‐A.

## 4 DISCUSSION

In this study, we investigated the potential role of Activin‐A, the distinct activator of heterotopic bone formation by activating R206H mutated ACVR1, on osteoclast formation in a recently established cell model for FOP (de Vries et al., 2018). In this model, we cocultured osteoclast precursors with PLF cells isolated from extracted teeth from controls and patients with FOP. These PLF cells come from a ligament, the kind of tissue that is being converted into bone in patients with FOP. After extraction of the teeth no heterotopic bone formation at the place of the extraction has been reported. Therefore, such tooth extractions can be regarded as a safe model to obtain primary cells from patients with FOP. Here we have refined the osteoclastogenesis cell model by using isolated CD14+ cells as a relatively pure source of osteoclast precursors to avoid possible interactions of Activin‐A with non‐osteoclast precursors present in peripheral blood.

QPCR results for ACVR1 and FKBP12 expression shows an altered responsiveness of the mutated receptor to Activin‐A. The upregulation of the inhibitor FKBP12 could be a response to the lost binding capacity of FKBP12 to the mutated receptor. The two‐fold upregulation of the ID‐1 expression in the FOP PLF monocultures suggests leaky signaling of the mutated receptor due to the inability of FKBP12 to bind to it (Song et al., 2010). Also, the higher ID‐1 expression in response to Activin‐A stimulation in the PLF cocultures shows the altered responsiveness of the mutated receptor. This response to Activin‐A was not seen in fibroblasts from control subjects. Recently Wang et al. (2017) reported that Activin‐A activates the SMAD 1/5/8 pathway in human primary FOP cells, using the tooth‐associated SHED cells retrieved from the primary dentition of patients. Here we show altered responsiveness of the ACVR1 receptor to Activin‐A in tooth‐associated PLF from patients with FOP.

Our results show that the PLF‐induced osteoclast formation of CD14+ cells was inhibited in the presence of Activin‐A. This inhibition was only significant in cocultures with FOP PLF, but this should be regarded with some caution, due to the relatively low numbers of control and FOP samples. The same trend was observed in controls. Considering the role of ACVR1 in osteoclastogenesis, the current study shows that activation of the receptor leads to less osteoclasts. Interestingly, deletion of the receptor, such as done in Osterix‐Cre mice, causes an increased osteoclast formation and alveolar bone with higher porosity (Zhang et al., 2018). Together, the current study and the study by Zhang et al. suggest a modulatory role of ACVR1 in osteoclast formation. Osteoclast formation in a coculture of PLF and osteoclast precursors is mediated by cell–cell interaction (Bloemen et al., 2011; Bloemen, Schoenmaker, de Vries, & Everts, 2010). This cell interaction, the binding of fibroblasts and osteoclast precursors by adhesion molecules, stimulates the production of osteoclastogenic factors such as M‐CSF and RANKL by fibroblasts, concomitant with osteoclast precursor expression of DC‐STAMP (Bloemen et al., 2010). In our coculture experiments, we observed a significant downregulation of M‐CSF and DC‐STAMP expression in the presence of Activin‐A, probably resulting in the observed decrease in osteoclast formation.

The current study demonstrated that the inhibition of osteoclast formation appears to be partially due to a direct effect on CD14+ cells. Activin‐A significantly inhibited osteoclast formation in CD14+ cells from healthy subjects. Even though M‐CSF was added to the cultures, there was a decrease in cell proliferation in cultures with Activin‐A, an observation also noted by Fuller, Bayley, and Chambers (2000) in a murine bone marrow cell culture system. The effect of Activin‐A on osteoclast formation and activity has thus far only been investigated in murine cells, and shows contradictory results, partly owing to the used population of osteoclast precursors. An enhanced osteoclast formation and activity was found in several studies using mouse total bone marrow or RAW264.7 cells as the source of osteoclast precursors (Fowler et al., 2015; Fuller et al., 2000; Gaddy‐Kurten, Coker, Abe, Jilka, & Manolagas, 2002; Kajita et al., 2018). However, Fowler et al. (2015) using bone marrow macrophages, showed a decrease in osteoclast formation due to decreased motility of the precursors as well as a decrease in osteoclast activity due to lower Cathepsin K expression and higher apoptosis. In our culture system we have used CD14+ cells as osteoclast precursors, a cell population that has some resemblance with the bone marrow macrophages used by Fowler et al. Using this culture system we observed a decreased osteoclast formation under the influence of Activin‐A, probably due to the diminished proliferation of the precursors.

As described above, the effects of Activin‐A on osteoclast formation in the coculture of PLF with CD14+ cells were similar to the effects found in the monoculture of CD14+ cells from healthy donors. This suggests that the effect seen in the cocultures is probably due to a direct effect on the CD14+ precursor cells. The observation that the inhibitory effect is significant when using the FOP fibroblasts, indicates that the mutated receptor might play an extra role in the osteoclast formation through a mechanism yet to be discovered. The lower M‐CSF and DC‐STAMP expression, molecules important in the proliferation and fusion of the osteoclast precursors, provide a potential mechanism for the decrease in osteoclastogenesis‐inducing potential of the FOP fibroblasts.

It has been described that Activin‐A induces bone formation in FOP by activating osteogenic differentiation through the mutated ACVR1 receptor. In this study we investigated the potential effect of Activin‐A on the osteoclast formation induced by PLF from patients with FOP, an issue that is all the more important because the first clinical trials using Activin‐a blocking antibodies are underway. Our study shows that, parallel to the already known positive effect on HO, the osteoclast formation and activity is reduced by Activin‐A in control CD14+ osteoclast precursors, and that the osteoclastogenesis‐inducing capacity of FOP PLF seems to be inhibited when exposed to this molecule. In line with our findings, Upadhyay et al. (2017) recently showed in their FOP mouse model that blocking Activin‐A with antibodies not only inhibited HO, but also resulted in partial resorption of already existing HO indicating that Activin‐A might be involved in tempering the recruitment or activation of osteoclasts. Taken together our data show that the inhibitory effect of Activin‐A on osteoclast formation is probably due to a direct effect on the CD14+ osteoclast precursors rather than an effect via the PLF. This indicates that blocking Activin‐A in FOP might block HO by both reducing bone formation as well as by increasing bone resorption. This adds another level of understanding to the regulation of bone formation in HO, the exploitation of which can lead to therapeutic gain.
